# Lossless Three-Dimensional Parallelization in Digitally Scanned Light-Sheet Fluorescence Microscopy

**DOI:** 10.1038/s41598-017-08113-8

**Published:** 2017-08-24

**Authors:** Kevin M. Dean, Reto Fiolka

**Affiliations:** 1 Lyda Hill Department of Bioinformatics, UT Southwestern Medical Center, 6000 Harry Hines Blvd., Dallas, TX 75390 USA; 2 Department of Cell Biology, UT Southwestern Medical Center, 6000 Harry Hines Blvd., Dallas, TX 75390 USA

## Abstract

We introduce a concept that enables parallelized three-dimensional imaging throughout large volumes with isotropic 300–350 nm resolution. By staggering high aspect ratio illumination beams laterally and axially within the depth of focus of a digitally scanned light-sheet fluorescence microscope (LSFM), multiple image planes can be simultaneously imaged with minimal cross-talk and light loss. We present a first demonstration of this concept for parallelized imaging by synthesizing two light-sheets with nonlinear Bessel beams and perform volumetric imaging of fluorescent beads and invasive breast cancer cells. This work demonstrates that in principle any digitally scanned LSFM can be parallelized in a lossless manner, enabling drastically faster volumetric image acquisition rates for a given sample brightness and detector technology.

## Introduction

Three-dimensional imaging of cells, tissues, and organisms requires increasingly fast, sensitive, and delicate microscopy platforms such that high-speed cellular events can be properly sampled in both space and time throughout large volumes^1^. Light-sheet fluorescence microscopy (LSFM) is an ideal candidate for imaging large volumes with high-resolution, high-sensitivity, and minimal phototoxicity^2, 3^. Commonly, in LSFM, the illumination light is injected into a sample at 90° relative to a detection objective and fluorescence originating from a single image plane is imaged with a scientific camera, delivering ~10^6^ greater parallelization than laser scanning confocal microscopy. Because the illumination is confined within the depth of focus of the detection objective, the resulting image contains less out-of-focus image blur, improved optical sectioning, and photodamage (e.g., phototoxicity and photobleaching) to the sample is minimized^4^.

In LSFM, volumetric data is collected by synchronously sweeping the light-sheet and detection objective in the Z-direction with a mirror galvanometer and piezoelectric actuator, respectively, or by scanning the specimen through a static light-sheet. Nevertheless, the serial acquisition of 2D images to form a 3D volume remains a bottleneck due to technological (actuator speed) as well as photophysical (finite photon flux) limitations^3, 5–11^. Further, methods that image multiple planes simultaneously (e.g., with diffractive or refractive optical elements), or extend the detection objective depth of field (e.g., by introducing aberrations or through point-spread function engineering), suffer from poor overall fluorescence collection efficiency and increased image blur, which obscures spatial detail^12–15^.

To mitigate these challenges, we developed parallelized Light-Sheet Fluorescence Microscopy (pLSFM), which provides cross-talk free imaging with three staggered light-sheets^16^. This enabled us to increase the volumetric acquisition rate to ~14 Hz, overcoming limitations in photon flux and piezoelectric actuator technology, without increasing the rate of photobleaching. However, pLSFM is restricted to imaging shallow volumes above a coverslip and is thus best suited for imaging thin adherent cells^16^. Further, due to geometrical constraints introduced by the coverslip and optical objectives, the numerical aperture (NA) of the detection objective in pLSFM is limited to ~0.8 (decreasing the photon collection efficiency and lateral resolution), and the light-sheets need to be axially separated by ~20–30 microns. As such, pLSFM is poorly suited for small specimens (e.g., yeast), cells within native tissue-like environments (e.g., synthetic hydrogels and extracellular matrix scaffolds^17^), or large specimens such as organoids, or model organisms.

Here, we expand upon pLSFM and present a general concept for parallelization of digitally scanned LSFMs^18^ with minimal cross-talk and light losses. Importantly, our method is capable of parallelized imaging of large volumes with no fundamental size restriction in any spatial dimension. Unlike pLSFM, the detection NA and the axial spacing of the light-sheets can be freely chosen (for a more detailed comparison, see Supplementary Note 1). Furthermore, the concept is compatible with existing digitally scanned light-sheet architectures. We present proof of concept by two-fold parallelization of a two-photon Bessel beam light-sheet microscope and present parallelized volumetric imaging of fluorescent nanospheres and invasive breast cancer cells^19^.

## Concept

**Figure 1:**
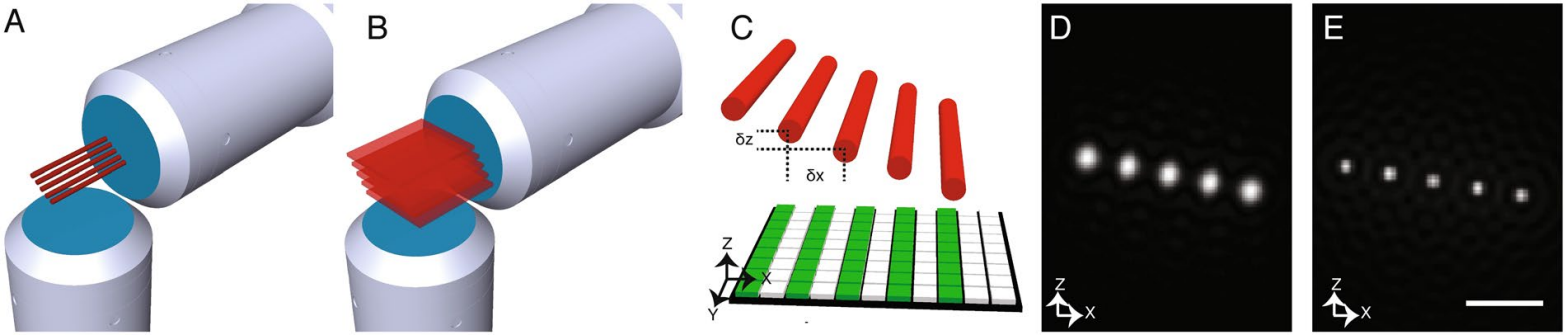
Optical concept. (**A**) Multiple 2D focused laser beams (e.g., 2-photon Bessel Beams), laterally and axially staggered by δx and δz, respectively, relative to the detection objective, illuminate the sample within the depth of focus of the detection objective. (**B**) Upon lateral scanning of the beam array, multiple light sheets are synthesized within the depth of focus. (**C**) The resulting fluorescence from each beam is detected on a camera (green stripes on a schematic pixel grid shown at the bottom), and each beam is independently resolvable at every scan position using camera based rolling shutters. (**D**) Simulation of a coherent superposition of Gaussian beams (NA = 0.1, λ = 488 nm). (**E**) Simulation of an incoherent superposition of nonlinear Bessel beams (Annulus ranging from NA = 0.63 to 0.64, λ = 900 nm). Scale bar 2 μm.

The general idea, presented in Fig. 1A, is to use multiple laterally and axially staggered (relative to the detection objective, e.g., in the X- and Z-dimensions) 2D illumination beams (Gaussian, Bessel, etc.). A single lateral scan of the illumination beams in the X-direction creates an array of synthetic light-sheets (Fig. 1B), each of which can be independently imaged with virtual confocal slit apertures (Fig. 1C)^20^. Because the beams are staggered and located within the depth of focus of the detection objective, the fluorescence arising from each illumination beam is in focus, achieves a high degree of optical sectioning, and is crosstalk free. To image a field of view spanning a distance *L* in the X-direction, with *n* beams, and an inter-beam spacing of $\delta x$, it is thus necessary to scan the beams a distance $L+n\delta x$, which is larger than the scan range normally employed with a single illumination beam (Figure S1). As such, the gain in volumetric imaging speed, *g*, for *n* spatially parallelized illumination beams is given by:

$$g=nL/(L+n\delta x)$$

Thus, if *L* ≫ *nδx*, the speed gain by parallelization approaches *n*, and the degree of parallelization is only limited by the number of beams that can be placed within the depth of focus of the detection objective.

To quantitatively evaluate this concept, we used numerical simulations and found that a coherent superposition of low NA (0.1 or smaller) Gaussian beams is relatively flexible – only small interference effects occur which are relatively insensitive to beam spacing^5^. Figure 1D shows simulations of a coherent superposition of five Gaussian beams, which is conveniently accomplished with diffractive optics^21^. However, because low-NA Gaussian beams have a large beam waist, these beams are best suited for low-NA detection objectives that have a large depth of focus, which is commonly the case for developmental imaging applications. The situation changes, however, when Bessel beams are superimposed coherently. As discovered by the Betzig lab, a coherent superposition of Bessel beams is very sensitive to the inter-beam spacing, which is leveraged in lattice light-sheet microscopy (LLSM) to sculpt the illumination field^3^. However, the side-lobe structures, as well as effects at the end of the lattice, make a coherent superposition of staggered 1-photon Bessel beams unattractive for 3D parallelization (Figure S2), especially when a field of view of ~100 microns is desired. Nevertheless, our simulations suggest that an incoherent superposition of 2-photon Bessel beams afford for very flexible beam spacing and drastically reduced side-lobe structures (Fig. 1E)^22^. Additionally, 2-photon Bessel beams maximize the field of view (~100 microns) while maintaining a narrow beam waist of ~370 nm, which enables multiple beams to be positioned within the depth of focus of a high-NA objective.

## Results

**Figure 2:**
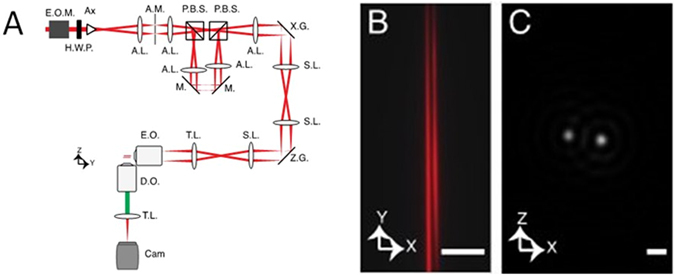
(**A**) Optical layout: E.O.M. electro-optic modulator, H.W.P. half-wave plate; Ax Axicon; A.M. Annulus mask; P.B.S. polarizing beam splitters (P.B.S.); A.L. Achormatic lens; M. Mirror; S.L. Scan lens; X.G. X-Galvanometer; Z.G. Z-Galvanometer; T.L. Tube lens; E.O. Excitation objective; D.O. Detection objective; Cam sCMOS camera. (**B**) Image of co-propagating, orthogonally polarized 2-photon Bessel beams resulting from excitation of a fluorescein solution. (**C**) Cross-section of co-propagating 2-photon Bessel beams. The squared intensity of the beams is shown. The Z separation of the two beams is 162 nm. Scale bar 1 μm.

As a proof of concept, we developed a light-sheet microscope that uses two staggered 2-photon Bessel beams to create ultrathin light-sheets^22, 23^. To avoid interference effects, the superposition of the beams was performed incoherently (the beams are orthogonally polarized and the beam splitting exceeds the coherence length of the light source). Figure 2A shows the optical layout. Ultrafast laser pulses from a Ti-sapphire oscillator are intensity modulated by a Pockels cell and shaped into a Bessel beam with an axicon. The Bessel beam is spatially filtered with an annulus mask in a 4f telescope. In the image space after the telescope, the Bessel beam is split into two separate beams with a pair of polarizing beam splitters, lenses, and mirrors. The two beams are scanned with an X- and Z-Galvanometers, which are relayed with three scan lenses and a tube lens to the back focal plane of the excitation objective (Nikon NA 0.8/40X water dipping). The resulting fluorescence is imaged at 90° in a light-sheet format with a detection objective (same type as excitation objective), tube lens, and a scientific complementary-metal-oxide-semiconductor (sCMOS) camera. Figure 2B shows the two Bessel beams as imaged with a water-fluorescein solution and Fig. 2C shows the squared intensity of the cross-sectional profile of the two beams. In our current implementation, an image was acquired with the sCMOS camera for each beam position in X, and the two light-sheet views were extracted in a post processing step^20^ using a moving virtual slit aperture with one pixel width. Preferably, each beam would be acquired with a multiplexed rolling shutter readout on a sCMOS camera, as is routinely performed with single beams^24^. However, this requires the development of new chip readout architectures, and is beyond the scope of this work. Additional mechanisms for image reconstruction are outlined in Supporting Note 2.

**Figure 3:**
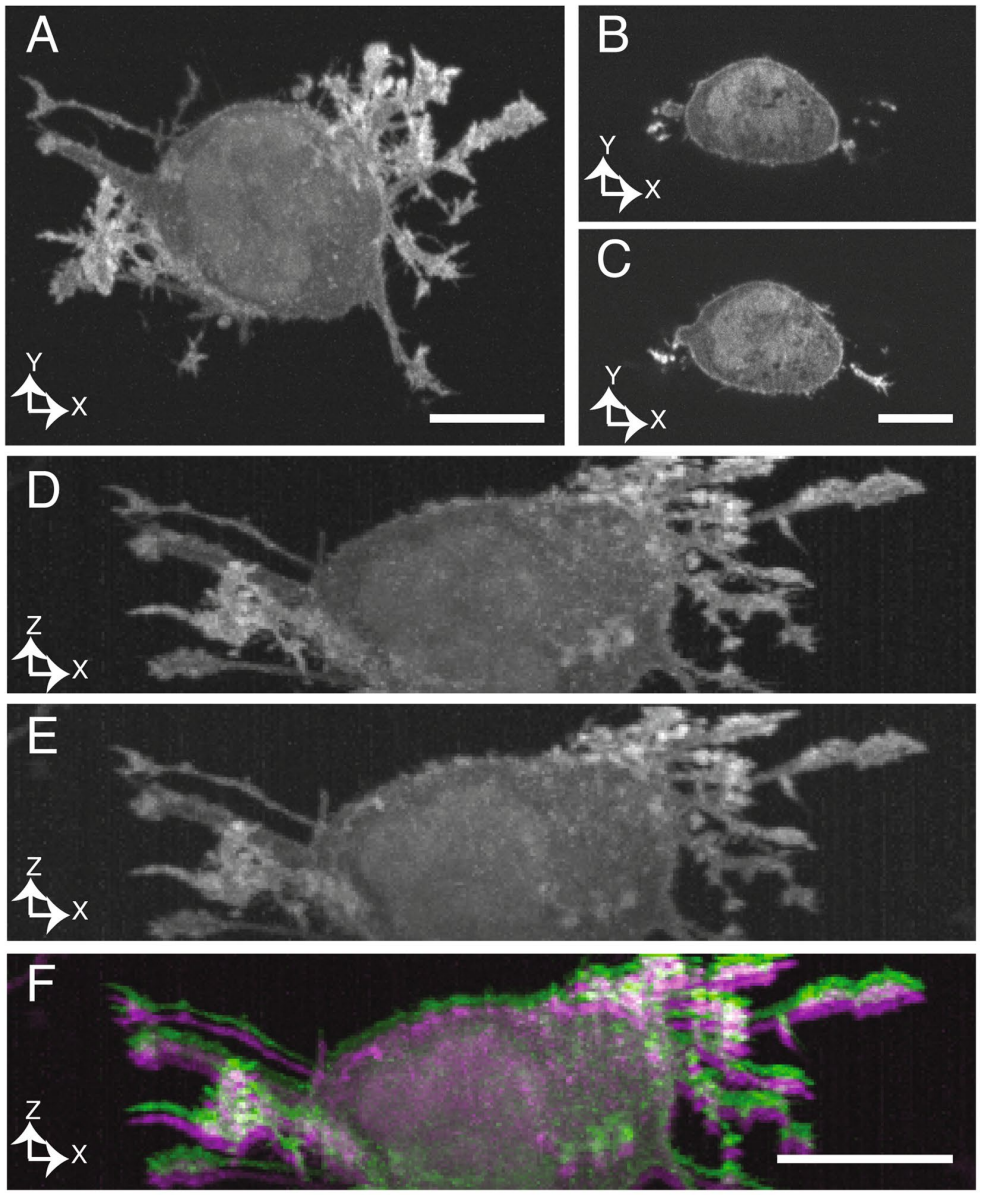
Parallelized imaging of collagen-embedded breast cancer cells. (**A**) Lateral maximum intensity projection, reconstructed from one of the illumination beams. (**B** and **C**) Single plane images acquired simultaneously with the two illumination beams, clearly showing different spatial features. (**D** and **E**) Axial maximum intensity projections resulting from simultaneous imaging with two illumination beams. (**F**) Overlay of two maximum intensity projections, showing 800 nm axial offset between the two image volumes. No deconvolution was applied to the shown datasets. Scale bars 10 μm.

To demonstrate that we can perform high-quality simultaneous two focal plane microscopy, we imaged an invasive population of breast cancer cells in a 4 mg/mL collagen I extracellular matrix^19^. Cells were labeled with AktPH, a GFP translocation biosensor that binds to phosphatidyl-inositol-3,4,5-trisphosphate (PIP_3_) and reports on local phosphoinositide-3-kinase (PI3K) activity, which is often aberrantly regulated in breast cancer. The axial spacing between the two beams was set to 800 nm, which is well within the depth of focus of our NA 0.8 detection objective (~2 microns, see Figure S3), but large enough to show a clear visual difference between the two focal planes. We acquired a 3D stack with an axial step size of 160 nm to obtain Nyquist sampled datasets of the cell from each illumination beam. Figure 3A shows a maximum intensity projection in the lateral dimension obtained with a single illumination beam. Figure 3B and C show individual XY cross-sections that were imaged simultaneously by the two beams. One can clearly see that the two beams imaged different parts of the cell, and that both images deliver an excellent level of detail, including filopodia and small organelles that were negatively stained by cytosolic fluorescence signal. Figure 3D and E show axial maximum intensity projections of image volumes obtained from each beam, both of which offer excellent optical sectioning and axial resolution. Figure 3F shows an overlay of the two axial maximum intensity projections, which are axially shifted to one another by the beam separation of 800 nm, but otherwise deliver precise correspondence of the imaged cellular structures.

**Figure 4:**
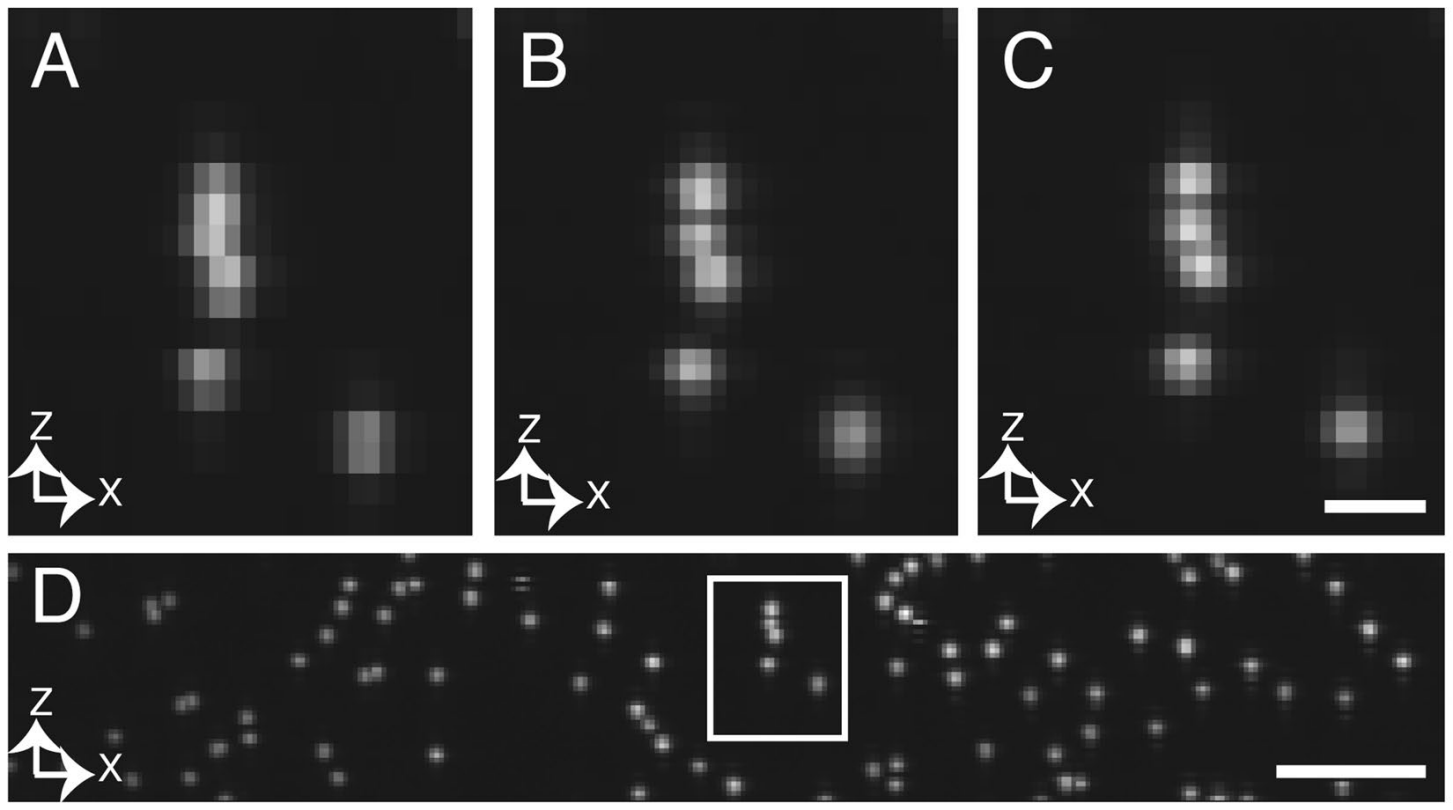
Interlaced imaging with sub-diffraction fluorescent nanospheres. (**A**) Axial maximum intensity projection of a single illumination beam with 320 nm axial sampling. (**B**) Axial maximum intensity projection of interlaced data resulting from two illumination beams, and 320 nm axial sampling. (**C**) Ground truth acquired with a single illumination beam and 160 nm axial sampling. Scale bar 1 μm. (**D**) Axial maximum intensity projection of interlaced data throughout a large field of view. Box indicates beads shown in panels (A–C). No deconvolution was applied to the datasets. Scale bar 5 μm.

Next, to demonstrate the potential of this method for parallelized imaging, we strategically separated the illumination beams axially by 162 nm and acquired a Z-stack of sub-diffraction nanospheres with a 320 nm step size. Because the axial resolution of our imaging system is ~350 nm, images generated by each beam were undersampled in the axial direction, resulting in clusters of beads that were not resolvable and isolated beads that were clearly under sampled in the Z-direction (see bottom right, Fig. 4A). However, by interlacing the data acquired from the two illumination beams, proper Nyquist sampling was restored, and individual beads became more clearly distinguished (Fig. 4B). Further, interlaced data were in good agreement with data acquired conventionally with proper axial sampling and a single illumination beam (Fig. 4C), and interlaced data quality was maintained throughout large field of views (Fig. 4D). Because the number of images acquired was decreased by the extent of parallelization (N = 2), the image stack was acquired ~2x faster than what would otherwise be possible with a single beam.

## Discussion

In summary, we have introduced a concept for 3D parallelization of digitally scanned light-sheet microscopes without introducing noticeable cross-talk or light losses. As proof of concept, we used two orthogonally polarized 2-photon Bessel beams that resided within the depth of focus and were imaged on the same camera. The coarse axial spacing used for the imaging of the breast cancer cells (800 nm) suggests that a larger number of beams with a smaller axial beam spacing could be used (e.g., 800 nm/160 nm = 5 intra-beam spacings for six beams). Moreover, according to our measurements of the depth of focus for our NA 0.8 objective (~2 microns) suggests that even greater parallelization is possible (Figure S3). In contrast to pLSFM (see Supporting Note 1), the beam spacing is flexible. We have shown fine control of the relative beam position using refractive optics, which allowed us to place the beams one z-step apart. We anticipate that for a coherent superposition of beams created by diffractive optics, rotation of the corresponding mask could be used to adjust the desired axial beam separation.

It is important to note that the general idea of staggering the beams in 3D space is not limited to narrow illumination beams or placing them within the depth of focus of the objective (See Figure S3, and Supporting Note 3). Therefore, we envision that this concept can also be applied to LSFM systems that image large, millimeter-sized volumes (e.g., drosophila embryos)^25^.

In summary, we believe that owing to its light efficiency, the presented concept for parallelization of LSFM will enable significant improvements in the volumetric acquisition rate while not compromising axial resolution, optical sectioning or sensitivity. This progress is needed as life scientists transition from optical coverslips to more complex, and more meaningful 3D environments, where speed, resolution, and sensitivity are critical imaging attributes for biological discovery.

## Methods

### Sample preparation

SUM159 cells were cultured in F12 base medium (Hyclone 5H30026.02) supplemented with 5% fetal bovine serum (Hyclone 5H30910.03), 100 units/mL penicillin-streptomycin (Hyclone 5V30010), 5 μg/mL bovine insulin (Sigma-Aldrich I1882), and 1 μg/mL hydrocortisone (Sigma-Aldrich H0135). Cells were infected with lentivirus (pLVX-IRES-Puro, Clontech) harboring the GFP-based biosensor, AktPH, and positively selected for with 1 μg/mL of puromycin (Sigma-Aldrich, P8833). For imaging, cells were trypsinized and placed into a pH-neutralized 4 mg/mL rat-tail collagen I solution (Corning, 354249), and the collagen was polymerized at 37 degrees Celsius in a custom polytetrafluoroethylene holder (All Axis Machining, Dallas, TX). Once polymerized, the sample was placed in culture media, incubated for 4 hours, fixed for 20 minutes at room-temperature with 4% paraformaldehyde in phosphate buffered saline, and imaged immediately. Sub-diffraction 200 nm beads (Polysciences Inc, 17151) were placed in a cubic block of 2% agarose.

### Image acquisition

The X galvanometer, Z galvanometer, Z objective piezo, Pockels cell, and Hamamatsu Flash 4.0 camera, were all controlled using custom software written in Matlab (Mathworks, Natick, MA) that included their data acquisition and image acquisition toolboxes. Analog voltages were delivered via two simultaneously operating USB-controlled data acquisition boards (USB-6001, National Instruments) and conditioned with scaling amplifier (SIM983, Stanford Research Systems). An image was acquired for each beam position in X, and the final image was reconstructed by extracting the actively illuminated pixels for each beam into separate image stacks. The total image acquisition time per plane was ~0.5 seconds. More information about the specifications of the microscope can be found in a previous publication^22^.

### Optical simulations

Simulations were performed in Matlab on a personal computer running Linux. Bessel beams were simulated by modeling their Fourier transform with a thin annulus (outer NA = 0.64, inner NA = 0.63), as described elsewhere^26^. A Fast Fourier Transform (FFT) of the simulated annulus yields the electric field in real space, which can be squared or raised to the fourth power to model the intensity distribution for a 1-photon and 2-photon illumination source, respectively. For the coherent superposition of Gaussian beams, a circular wavefront was passed through an ideal lens with a numerical aperture of 0.1 and the field was forward propagated numerically to the focal plane. For the coherent superposition, shifted copies of the in-focus electric fields were summed. To obtain the intensity distribution, the squared modulus of the resulting electric field was taken. Coherent lattices of Bessel beams were performed for 1- and 2-photon illumination using the square, or ‘sensitivity’ mode^3^ for a beam propagation length of 100 microns. In a separate simulation, the beam spacing was doubled, which is no longer a square lattice, as it contains additional diffraction orders in the back-pupil plane.

## Electronic supplementary material

Supplementary Materials
