# Supplementary material for: Lossless Three-Dimensional Parallelization in Digitally Scanned Light-Sheet Fluorescence Microscopy
Source: Sci Rep. 2017 Aug 24;7:9332. doi: 10.1038/s41598-017-08113-8 (PMC5570909; doi:10.1038/s41598-017-08113-8)
Supplement: Supplementary file 1 — Supplementary Materials [file 41598_2017_8113_MOESM1_ESM.pdf]

Lossless Three-Dimensional Parallelization in Digitally Scanned Light-Sheet Fluorescence Microscopy

Kevin M. Dean[1] and Reto Fiolka[2*]

[1]Lyda Hill Department of Bioinformatics, UT Southwestern Medical Center, 6000 Harry Hines Blvd., Dallas TX, USA, 75390.

[2]Department of Cell Biology, UT Southwestern Medical Center, 6000 Harry Hines Blvd., Dallas TX, USA, 75390

*Corresponding author: reto.fiolka@utsouthwestern.edu

Supplementary Materials

Supporting Figures

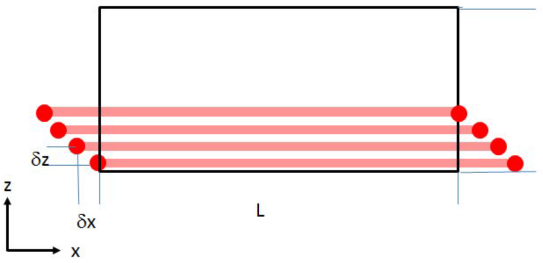

Fig. S1. Parallelized acquisition of a cuboid imaging volume with a length L in the scan direction of the beams. Since the beams are staggered, the scan range has to exceed the volume such that all beams cover the desired lateral field of view.

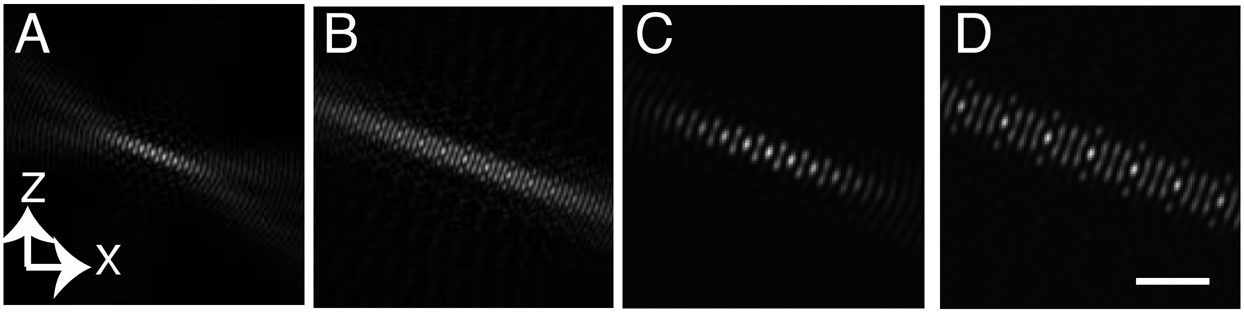

Fig. S2. Coherent lattice light-sheet illumination with a beam propagation length of 100 microns. In each case, the upper NA was 0.64. (A) 1-Photon square lattice with normal and (B) doubled beam spacing. (C) 2-Photon square lattice with normal and (D) doubled beam spacing. In each case, the coherent illumination results in broadened intensity distribution in the Z direction, and no clear interface between beamlets in the X direction, rendering it incompatible with the parallelized scheme reported here. Scale bar, 5 microns.

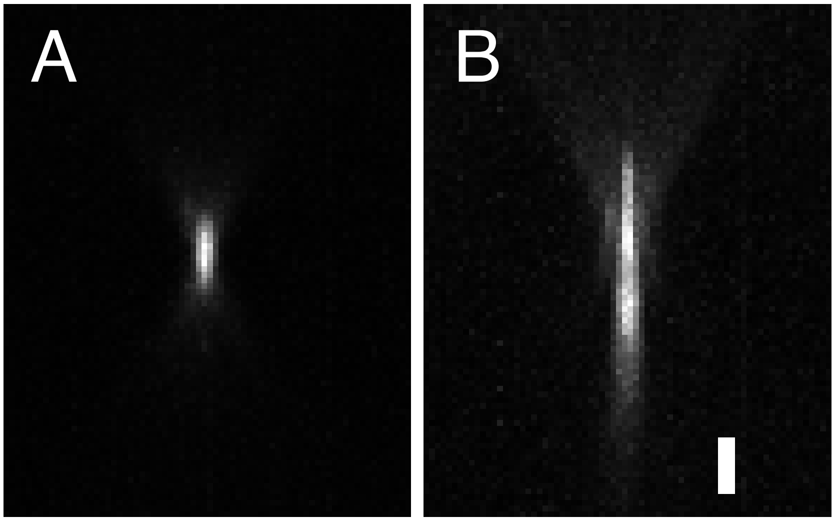

Fig. S3. Axial Point spread function of a 40x NA=0.8 Nikon objective before and after adding aberrations. Aberrations were introduced with a deformable mirror conjugate to the back-pupil plane of the detection objective. (A) Aberration-free PSF with a confocal parameter of ~2 microns in the axial direction. (B) Spherically aberrated PSF with a confocal parameter of 3.6 microns. The spherical aberrations extend the depth of focus by ~1.8x, but the Strehl ratio decreases by a factor of ~3.1. Scale bar 2 microns.

Supporting Note 1 - Comparison with pLSFM

The microscope presented here employs staggered beams to parallelize imaging in the Z-direction, which at first glance, appears conceptually similar to pLSFM.[16] However, many conceptual and practical differences exist. pLSFM is designed for thin, adherent cells, opportunistically positioned 45 degrees relative to the excitation and detection objectives. It uses static (e.g., non-scanned) one-dimensional light-sheets that are staggered axially and laterally (*relative to both the excitation and detection objectives*) so that the beam waists coincide with the optical coverslip and form images that can be separated in 3D image space without light loss and cross talk. In pLSFM, the spacing of the beams was dictated by the opening angle of the coverslip, NA of the detection objective (i.e. its opening angle), and the ability for each beams fluorescence emission to be efficiently picked-off by the knife-edge prism. These factors restricted pLSFM to a maximum NA of 0.8, a beam spacing of ~25 microns, a field of view of ~13 microns in the beam propagation direction, necessitated deconvolution to remove spherical aberrations from two of the three views, and was best suited for thin and elongated specimens (> 50 microns). Increases in the field of view could only be achieved by increasing the beam spacing, which increases aberrations, or by reducing the NA, which decreases resolution and sensitivity.

In contrast, the optical concept presented here shares none of the aforementioned limitations. The field of view, beam spacing and detection NA can all be independently adjusted. It staggers high-NA two-dimensional pencil-like beams (Gaussian, Bessel, etc.) laterally and axially (*relative to the detection objective, only laterally relative to the excitation objective*), and synthesizes multiple virtual light-sheets by laterally scanning the beams. Because each virtual light-sheet resides within the depth of focus of the detection objective, the resulting fluorescence is in focus and deconvolution is not necessary (although it can be used to further improve the resolution). Unlike pLSFM, the size of the volume that is being imaged can be adjusted to accommodate any 3D sample within the limits of our galvanometers, scan optics, and objective piezo. The experiments presented here were performed over a lateral field of view (80x80 μm^2) that is ~6-fold larger than the critically limited dimension in pLSFM. The inter-beam spacing was 800 and 160 nm, representing 30x and 156x finer positioning than what is possible with pLSFM, respectively. This enables imaging of correspondingly smaller samples and volumes with fully parallelized detection, which would appear in pLSFM only on a single image plane. Further, the axial resolution, even without deconvolution, is 1.7-fold better than pLSFM. However, due to limitations in camera technology, only two beams were implemented. In the future, we envision that sufficient technological developments should allow for 8-fold parallelization with a NA 1.1 water dipping objective, which has a depth of focus of ~1.2 microns[3] (7x0.16 = 1.12 microns).

**Optical Parameter**	**pLSFM (Optica, 2017)**	**This Manuscript**	**Fold Improvement**
Beam Spacing	25 μm	160-800 nm	Up to 156x
Parallelization	3x	2x as presented, up to 8x.[α]	Up to 2.66x.
Number of Cameras	3	1	3x
Maximum Detection NA	0.1-0.8	0.1-1.1	Up to 2[†]
Resolution (x,y,z)	300, 300, 600 nm	300, 300, 340 nm	1.7x, axially.
Field of View in Y	13 μm	80 μm	6.15x

[α] Depth of focus of 1.2 microns, as reported for the best of class Nikon 25x NA=1.1 water dipping objective. Actual experiments used a Nikon 40x NA=0.8 water dipping objective.

[†] In detected photons, an NA 1.1 objective has ~twofold larger solid angle than an NA 0.8 objective, thus the collection efficiency is doubled

Supporting Note 2 - Methods for Image Acquisition

Here we present and discuss several methods for image acquisition using axially and laterally staggered illumination beams.

Sample Scanning

In the simplest case, a sample is illuminated with N-beams axially and laterally staggered within the depth of focus of the detection objective, and N-beams are detected within a region of interest on a camera. The sample is scanned in the X-direction by a step size of one pixel, and an image of all N beams is acquired simultaneously. This process is repeated for a distance L+N•δX, building up the field of view. The sample is then stepped in the Z-direction by N•δZ and the process is repeated. The decreased size of the camera region of interest affords short readout times and high framerate imaging, so long as rapid sample repositioning does not disrupt the specimen. The illumination train is massively simplified since scan optics, galvanometers, and objective piezos are not required. Nevertheless, sample repositioning with piezoelectric stages will be significantly slower than what is possible with galvanometer-based beam scanning. In principle, this method is also compatible with pixel reassignment, which would provide super-resolution in the X-direction.[27]

Stepwise Imaging

Alternatively, the sample remains stationary, the beam array is scanned by 1 pixel in the X-direction, an image is acquired at each beam position and the process is repeated over the scan range L+N•δX. The beams are then stepped in the Z-direction by N•δZ and the process is repeated. The final image volume is reconstructed by computationally applying rolling confocal apertures, and pixel reassignment can also be used to achieve super-resolution in the X-direction. However, the full field of view is acquired for each beam position, which necessitates slower readout times.

Parallelized Light-Sheet Readout

The most elegant solution is to parallelize the light-sheet readout mode of a single CMOS camera. Here, N-beams are swept across the camera, and N regions of active pixels are swept across the camera synchronously. Each active pixel region is assigned its own image plane, and all N-beams, and thus all N-image planes, are acquired in a single lateral scan. The illumination beams, and the detection objective, are then stepped in the Z direction by N•δZ and the process is repeated. For a Hamamatsu Flash 4.0 camera, we anticipate that the maximum framerate possible using this mode, should it become available, would be ~49N and ~196N frames per second, for a 2048x2048 and a 2048x512 pixel image size, respectively. For an 80x80x80 μm^3 volume, sampled with a 160 nm Z-step size, and imaged with 6 illumination beams, this would result in a 2.3 Hz volumetric image acquisition rate.

Fluorescence Descanning

The most complex imaging mechanism would involve physically descanning the fluorescence emission. Here, one would place a galvanometer in an image plane that is conjugate to the back pupil of the detection objective, and synchronize this galvanometer with the X-galvanometer that is located within the illumination optical train. However, because most high-NA objectives have a back-pupil plane that is located within the objective housing, a 4f imaging system is required to relay the back pupil of the objective to the galvanometer, and an additional lens is required after the galvanometer to image the now static beams onto a camera or series of line cameras. If using a single camera, then separate regions of interest could be used to reconstruct the image from each illumination beam. In each case, very high bandwidth cameras are necessary.

Supporting Note 3 - Imaging outside of the depth of focus

In its simplest implementation, all illumination light-sheets remain within the depth of focus of the detection system to yield sharp images on a single camera. Typical high-resolution light-sheet microscopes use numerical apertures in the range of NA=0.8 to NA=1.1. For our NA=0.8 objective, we measured a confocal parameter of ~2 microns in the axial direction of the PSF and the depth of focus for an NA=1.1 microscope ranges practically within 1.2 microns.[3] To increase the axial range over which light sheets can be placed, various concepts exist to extend the depth of focus. They can be categorized into either static wavefront engineering using phase masks or rapid defocusing using tunable lenses or deformable mirrors. Wavefront engineering will inevitably lower the Strehl ratio of the system, as the PSF is stretched and the contained intensity is spread over a larger volume, and likely lateral resolution will be lowered as well. Rapid refocusing, even if spherical aberrations are compensated, will lower the collection efficiency, as any focal plane is only in focus for a short time. For parallelized light sheet imaging presented here, in contrast to previous use of extended focusing in light sheet microscopy, only modest increases in the depth of focus are needed. As an example, we extended the confocal parameter of our detection PSF to 3.6 microns by introducing spherical aberrations with a deformable mirror conjugate to the back pupil of the objective. Nevertheless, this decreased the Strehl ratio by ~3.1-fold.

Supporting References

3 Chen, B. C. *et al.* Lattice light-sheet microscopy: Imaging molecules to embryos at high spatiotemporal resolution. *Science* **346**, 1257998-1257998, doi:10.1126/science.1257998 (2014).

16 Dean, K. M. *et al.* Imaging subcellular dynamics with fast and light-efficient volumetrically parallelized microscopy. *Optica* **4**, 263, doi:10.1364/optica.4.000263 (2017).

27 Sheppard, C. J. R., Mehta, S. B. & Heintzmann, R. Superresolution by image scanning microscopy using pixel reassignment. *Opt. Lett.* **38**, 2889, doi:10.1364/ol.38.002889 (2013).
